# Comparison between Tests and Simulations Regarding Bending Resistance of 3D Printed PLA Structures

**DOI:** 10.3390/polym13244371

**Published:** 2021-12-14

**Authors:** Dorin-Ioan Catana, Mihai-Alin Pop, Denisa-Iulia Brus

**Affiliations:** 1 Department of Materials Engineering and Welding, Transilvania University of Brasov, 500036 Brasov, Romania; 2 Department of Materials Science, Transilvania University of Brasov, 500036 Brasov, Romania; mihai.pop@unitbv.ro; 3 Department of Motor Performance, Transilvania University of Brasov, 500036 Brasov, Romania; denisa.brus@unitbv.ro

**Keywords:** additive manufacturing, poly(lactic acid), optimization, simulation, finite element analysis (FEA)

## Abstract

Additive manufacturing is one of the technologies that is beginning to be used in new fields of parts production, but it is also a technology that is constantly evolving, due to the advances made by researchers and printing equipment. The paper presents how, by using the simulation process, the geometry of the 3D printed structures from PLA and PLA-Glass was optimized at the bending stress. The optimization aimed to reduce the consumption of filament (material) simultaneously with an increase in the bending resistance. In addition, this paper demonstrates that the simulation process can only be applied with good results to 3D printed structures when their mechanical properties are known. The inconsistency of printing process parameters makes the 3D printed structures not homogeneous and, consequently, the occurrence of errors between the test results and those of simulations become natural and acceptable. The mechanical properties depend on the values of the printing process parameters and the printing equipment because, in the case of 3D printing, it is necessary for each combination of parameters to determine their mechanical properties through specific tests.

## 1. Introduction

The twentieth century was marked by the unprecedented development of the engineering sciences. This evolution was possible due to the important steps made in the theoretical field by other disciplines, including mathematics, physics, and chemistry. The demonstration of some basic theories and theorems in mathematics and physics, as well as the inclusion of mathematical analysis and differential equations in the solution of engineering problems, meant finding theoretical solutions to practical aspects encountered in engineering. The end of the previous century allowed the development of technologies and equipment for 3D printing. Additive processing is a process of continuous exploitation and exploration. Exploitation because new and new applications in different fields are solved using this technology, and exploration because researchers attempt to find solutions to the hitherto unresolved aspects of this technology, which, as research shows are not few.

Additive manufacturing has seen the rapid expansion and continuous development of printable materials. 3D printing is currently widespread and it has begun to address new areas through its involvement in different forms of goods manufacturing and in sports (such as alpine skiing). Given the relatively short time in which that 3D printing has been used, this technology still features many aspects that need to be understood, solved, and improved. Currently, producers want to reduce the time it takes for products to reach the market and for consumers (beneficiaries) to purchase them at the lowest possible cost. The way to improve the properties of 3D printed structures is through the constant attention of researchers, who attempt to apply different techniques to reach the proposed objectives. This approach consists in applying the capabilities of the simulation process to 3D printed parts, to optimize the respective products. Bibliographic study demonstrates that currently, there are few works that address the implementation of the simulation (modelling) process, with its undeniable benefits, in additive manufacturing.

The printing parameters can significantly influence the mechanical properties of the 3D printing parts (printing speed and nozzle temperatures). Furthermore, with a finite element analysis (FEA) the stress distribution of single-tensile testing, bending testing, and compression testing of poly(lactic acid)-PLA samples has been visualized [1]. The simulation can provide critical inputs for the designer. Moreover, based on experimental data obtained (extracted) from previous research, a finite element analysis can be applied. Studies reveal that the deviations between simulation and experimental results were minimal, and the maximum error was 6.7%. In this way, the simulation could be used to predict the behaviour of 3D printed parts [2]. Experimental and theoretical results demonstrate that the tensile strength of 3D printed poly(lactic acid) decrease as the layer thickness increases from 0.1 to 0.3 mm. Furthermore, the experimental results demonstrate that the ultimate tensile strength of 3D printed samples changes significantly with changes in the printing angle [3]. Simulation results show that strain and displacement in the gage region offer results that are comparable with experimental results [4]. The improvement of the 3D printed part quality requires many studies about the optimal setting for additive manufacturing parameters [5]. Research shows that 3D printing parameters significantly influence the elastic strength of polymer composites [6]. The best results from the tests and simulation were obtained when the infill pattern was 100%. In addition, the difference between the experimental results and the simulation was below 10% [7,8]. With a low filament consumption, the researchers showed that the values of the printing parameters can be optimized in the case of additive manufacturing, based on material extrusion [9]. The simulation can be applied to predict the behaviour of the 3D printed structures, from PLA [10].

The study of the published articles on the additive manufacturing of poly(lactic acid) led to the following conclusions:

- material is assimilated as homogenous and linear-elastic [11];
- anisotropy of 3D printed structures is mild [12];
- Poisson’s ratio for the 3D printed parts is between 0.33–0.36.

All this information highlights that there are necessary bases for the application of the simulation process for the PLA structures, obtained by additive manufacturing. Furthermore, Refs. [13,14] demonstrate that the simulation process can be applied with good results to the 3D printed structures from PLA, because the differences between the test results and those of the simulation feature reduced errors. The simulations were performed based on the results obtained from the tensile and bending tests performed on the 3D printed structures from PLA. In previous studies [13,14], the mechanical properties of the 3D printed structures were determined from the used filaments, because there were significant differences between the properties of the filaments and those of the printed structures. It should be noted that 3D printing involves the filament melting, followed by its solidification. Thus, the PLA obtained structures by additive manufacturing consist in solidifications of the deposited filament, in successive layers. This approach to generating 3D printed structures influences their resistance to different stresses (loadings). The explanation is that between the successively deposited layers (one already solidified and the other in the process of solidification), strains appear, which can reduce, to a greater or lesser extent, the properties established by the calculus. The reason why 3D printed structures need to be tested is to determine their mechanical properties. When the mechanical properties are determined, they can be passed to the optimization stage by simulation.

Poly(lactic acid) was chosen because it is one of the most commonly used filaments in additive manufacturing, and it also features many applications in the medical field. In addition, the tensile and bending stresses were studied because these are the stresses that develop most often in the 3D printed structures from PLA. The aim of the paper is to demonstrate that, based on existing or determined information regarding the physical and mechanical properties of 3D printing, finite element analysis (FEA or FEM-finite element modelling) can be applied to optimize the respective structure from the geometric and dimensional point of view. The results of the simulation process, which are in line with those of theory and previous tests, make it possible to reduce the design time, while improving the behaviour at various stress points for 3D printed structures.

## 2. Experimental Setup

The materials (filaments) used in the specimen printing were poly(lactic acid) (PLA white manufactured by Suntem3D, Bucharest, Romania) and poly(lactic acid) mixed up to 20% with glass fibre (PLA-Glass, manufactured by Filaticum, Miskolc, Hungary). For the PLA, the mechanical properties included: filament diameter 2.85 mm, tensile strength 1100 MPa (ASTM D882), modulus of elasticity 3310 MPa (AST MD882), and bending modulus of elasticity 2392.5 MPa. For the PLA-Glass, the mechanical properties included: filament diameter 2.85 mm, maximum tensile strength 57 MPa (ASTM D638), tensile strength at yield 46 MPa (ASTM D638), tensile modulus 4.0 GPA (ASTM D638), and tensile elongation 3.4% (ASTM D638). The complete technical characteristics of both filaments are presented in their respective technical data sheets.

From the mentioned filaments, the 3D specimens to be used in the bending tests were printed, in order to optimize them from a geometric and dimensional point of view. For the 3D printing parameters with which the PLA structures were processed, the mechanical properties were determined and presented in previous works [13,14]. In the case of additive manufactured structures, for the simulation process to be performed, the mechanical properties necessary are the following:

- density of the 3D printed specimen; this is not equal with the density of the filament, because it depends on the 3D structure’s printed parameters;
- bending deflection;
- yield strength;
- modulus of elasticity;
- ultimate strength at bending (bending strength);
- Poisson’s ratio.

The printing of the specimens performed on a CreatBot DX-3D double-nozzle printer (manufacturer Henan Suwei Electronic Technology Co., LTD., Zhengzhou, China). The printer capabilities were:

- printing dimensions—300 × 250 × 300 mm;
- filament diameter—2.8–3.0 mm;
- printing nozzle—0.2–0.8 mm;
- printing resolution—0.6 mm;
- layer resolution—0.2 mm;
- printing volume—22.5 l.

The parameters of the printing process were:

- layer height—0.2 mm;
- printing temperature—210 °C;
- print speed—50 mm/s;
- printing angle (overhang angle for support)—45°;
- bed temperature—61 °C;
- infill—100% (the internal structure is solid; with a solid infill at the top and bottom);
- infill overlap—10%;
- infill flow—110%.

The printed structures from the PLA are of bar or tube type, with circular, elliptical or rectangular sections. To verify the efficiency of the simulation process, specimens with a profile I section were printed. These specimens were bar type. Furthermore, tube type specimens with rectangular sections, but consolidated in the middle, were produced (cross-section Rect-cons). Additive manufacturing specimens were obtained from the PLA filament (100% PLA) or PLA-Glass filament (100% PLA-Glass). Table 1 presents the coding of the specimens according to the material, the geometry of the section, the dimensions, and the type.

**Figure 1:**
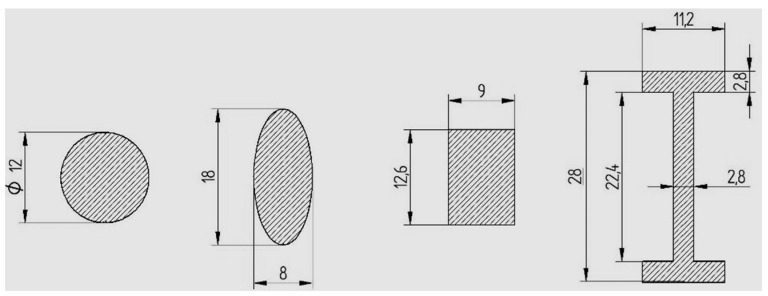
Shapes and dimensions of the bar-type specimens (ISO metric drawing standard).

**Figure 2:**
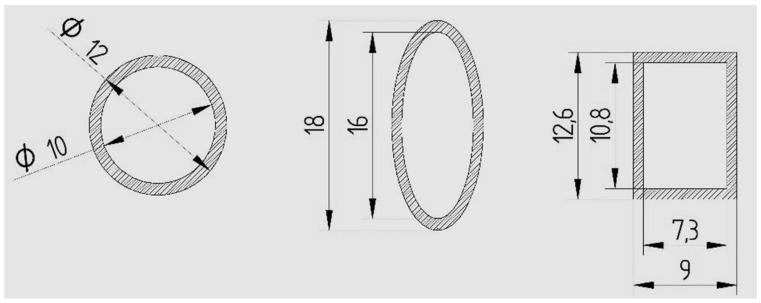
Shapes and dimensions of the tube-type specimens (ISO metric drawing standard).

The shapes and dimensions of the specimens are presented in Figure 1 and Figure 2.

The 3D printed specimens were tested at bending and the equipment used was a WDW-150S Universal testing machine. The bending tests were performed under the following conditions:

- bending (loading) speed 10 mm/min;
- stress speed 10 MPa/s;
- support—cylindrical (diameter 30 mm, length 70 mm);
- loading nose/anvil—semi-cylindrical (diameter 30 mm, length 70 mm).

On this testing machine, the test force can be modified between 0.1 and 150 kN.

Due to the bending stress, the straight beams (girder) become deformed and curve-shaped until they exceed a critical value, at which point they break. Bending tests allow the bending strength and deformation (displacement) to be obtained. Deformation, also known as the arrow, represents the maximum transverse displacement produced in the middle of the opening of a beam supported at its ends. The value of the deformation represents the shape that the beam can form when it is bent under stress or the deformations produced near some sections. The bending tests made it possible to determinate the maximum force that produced the breaking (rupture) of the specimens and, based on this, it was possible to calculate the bending strength. The tests were performed for both the printed PLA and the PLA-Glass specimens. The tests were also performed for the bar- or tube-type specimens and for the geometries displayed in Table 1. By complementing the information established during other tests and available in various papers with those obtained during the tests presented in the paper, the simulation process can be started.

Before presenting and discussing the results of the simulation, it should be noted that regardless of the geometry of the cross-section, the value of the section is the same for the bar specimens (113 mm^2^). The tube type specimens also feature the same value for the respective section, but of course, the value is significantly reduced (34.5 mm^2^, which is 31% of the bar type section’s value). In this way, the efficiency of each type of specimen and geometry can be better understood, while comparisons between results can be made more easily. The bending force was applied halfway between the supports, where the deformation of the specimen was maximal. Based on the test results, the simulation process could be applied, which was performed under the same conditions as the tests, which were as follows:

- the stress (load) force was equal to the one during the tests;
- the force was applied halfway between the supports;
- the distance between the supports was 180 mm;
- the physical and mechanical properties of the 3D printed structures were those established by the tests, previously presented.

For the geometry of the section, we opted for the shapes described in Figure 1 and Figure 2. According to the theory, the bending strength depends on the moment of inertia (I_z_) and the distance between the point on the surface for which the calculus is made and the neutral axis (y_max_). The ratio between I_z_ and y_max_ is called the axial resistance moment (W_z_) and is a geometric feature of the cross section. The bending strength for the described stress scheme is calculated by the relation:

(1) $$\sigma_{\max}=\frac{M_{i}\cdot y_{\max}}{I_{z}}=\frac{M_{i}}{W_{z}}=\frac{F_{\max}\cdot l}{4\cdot W_{z}}\;,$$

where M_i_ is the bending moment, W_z_ the axial resistance moment (modulus), F_max_ the maximum force that produced the rupture of the specimen, and l is the distance between the supports. The study of relation (1) shows that in order to obtain the lowest possible bending resistance, the geometry of the structure must feature an axial resistance modulus that is as large as possible because it is assumed that the distance between the supports is kept constant. In addition to the bar-type specimens, tube-type specimens with the same cross-sectional geometries as those of the filled section (bar) specimens were printed.

The finite element analysis was performed with the Simulation module in Solid Edge ST10 2D and 3D software for engineers (Siemens Industry Software Inc., Plano, TX, USA). The simulation process was performed under the following conditions: mesh type–tetrahedral; study–linear static; meshing level–9 for all simulations, which generated a mesh size between 1.6 and 3.45 mm (depending on specimens’ type and their geometries).

## 3. Results and Discussion

The specimens obtained through additive manufacturing were tested for bending. As mentioned, this stress (load) is common in 3D printed structures. The simulation results are presented in Table 2.

**Figure 3:**
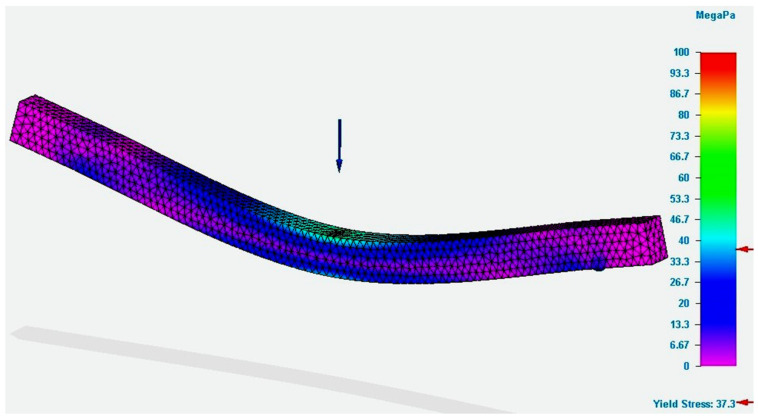
Results of the simulation process for P_R12 specimen (Von Mises stress).

Figure 3 depicts the simulation results for the specimen with the rectangular section obtained from the PLA filament.

**Figure 4:**
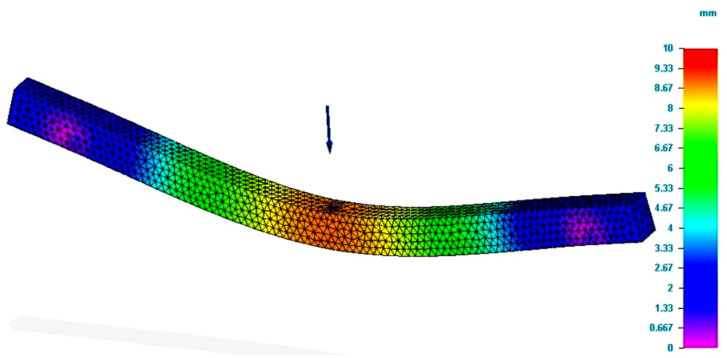
Results of simulation process for P_R12 specimen (total deformation values).

The figure presents the value of the Von Mises stresses developed in the specimen, when a force equal to that obtained in the bending tests was applied. In the force application area, the figure demonstrates that the value of the bending stress was close to that determined by tests. The value determined by the simulation was smaller than the one registered in the test. Regarding the deformation value (see Figure 4), it was found that it was greater than the real value, namely, the value recorded by the test equipment. Regarding the deformation, it should be mentioned that the theory demonstrates that the analytical method of integration of the differential equation is applied for an approximate deformed average fiber. Therefore, there are possible errors in the calculus compared to reality.

**Figure 5:**
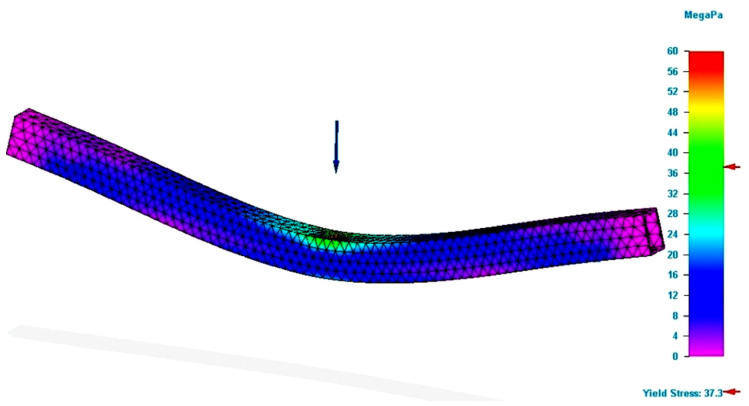
Results of simulation process for P_R12_T specimen (Von Mises stress).

In Figure 5, the simulation results for the tube-type specimen with rectangular section obtained from PLA filament is presented.

**Figure 6:**
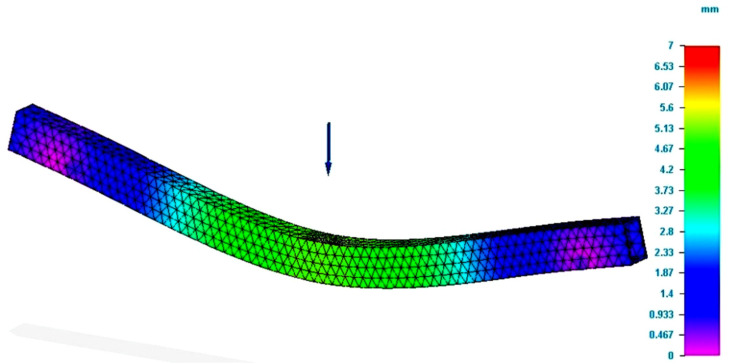
Results of simulation process for P_R12_T specimen (total deformation values).

The simulation of the deformation for the specimen P_R12_T is presented in Figure 6. Using the capabilities of the simulation program, the value of the maximum deformation that occurs in the specimen before it ruptures can be determined.

The value obtained by the simulation was greater than the value recorded during the test. This error can be explained by the approximate methods of calculating the average fibre, but also by the elasticity of the tube-type specimen.

Analyzing Table 2, it can be seen that the application of the simulation provided results close to those of the tests. In addition, the test and simulation data demonstrate that it was possible to optimize the geometry of the 3D printed structures. This statement is based on the following aspects. As mentioned, the bar-type specimens featured equal sections, as did the tube-type specimens. The length was 220 mm for all the 3D printed specimens. The deduction is that the volume was the same (V = A·l) for the bar or tube-type specimens, including their mass. All this occurred because it depended on the volume and density (m = Vρ). The density of the specimens remained constant if the printing parameters were not changed.

Therefore, the efficiency of the section geometry is given by the ratio between the axial resistance modulus and the surface. When the W_z_ is higher, for the same surface of the 3D printed structure, the specimen can be stressed with higher forces until breaking. Thus, the ratio between the axial resistance modulus and the area can be considered an indicator of the efficiency of the section geometry, or it can reveal whether the section is optimal. Therefore, as this ratio is greater, the volume of the printed material in this structure is smaller, but with the same bending strength, or even higher.

For the validation of the presented features, structures with section I were printed by additive manufacturing. The respective structure featured a cross section 11% larger than the bar-type structures used in the study. The dimensions of the surface made it possible to increase the height of the specimen, which had a significant impact on the ratio between the breaking force and the volume (see the last column in Table 3).

**Figure 7:**
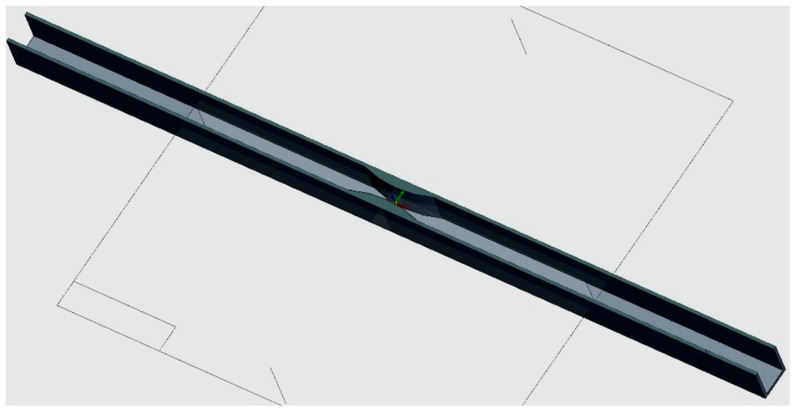
Rectangular specimen with consolidation (inside view).

Furthermore, in the case of the rectangular specimens with the tube section, a consolidation was performed in the middle of the specimen (see Figure 7). The efficiency of this consolidation can be followed by studying the values in the last column of Table 3. The comment that needs to be made is that the value of the section is valid only for the middle of the specimen, where it decreases by half, reaching the value of the tube-type sections. The application of the consolidation demonstrates that the force that caused the specimen rupture was greater than the force at which the specimens broke without this modification.

**Figure 8:**
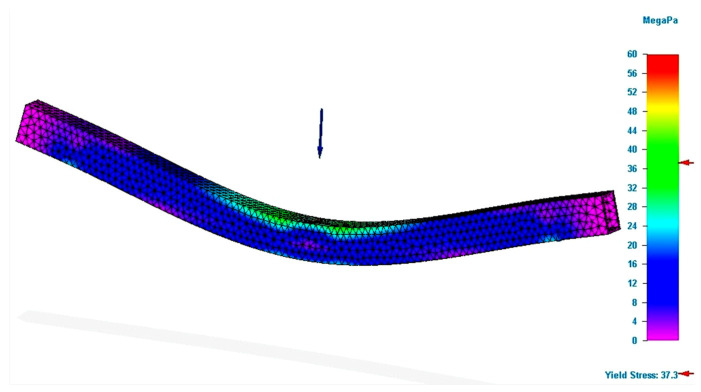
Results of simulation process for P_RC_T specimen (Von Mises stress).

The positive evolution is valid for both materials used in the study. From the point of view of volume, its increase by 6.5% for the reinforced rectangular specimens determined an increase in the breaking force by 34.80% for the P_RC_T specimens and by 10.4% for the G_RC_T specimens. In other words, with a small addition of material in well-defined places, the bending strength increases (see Figure 8).

Analyzing Figure 5 and Figure 8, the differences between the stresses generated in the same zone of the specimen are clearly demonstrated. This evolution is favorable for the specimens that benefited from consolidation. Thus, the comparison demonstrates that the simulation process can be applied with confidence in the additive manufacturing of PLA structures and can be considered a useful and important tool for designers. The presented features demonstrate that by optimizing the geometries of the additive manufactured structures using the simulation process, it is possible to substantially improve their behavior at the stresses that are applied to them. A smaller volume of printed filament also means lower energy consumption for the structure processing. Implicitly, at the end of the lifecycle of the structure, the volume to be recycled is lower.

**Figure 9:**
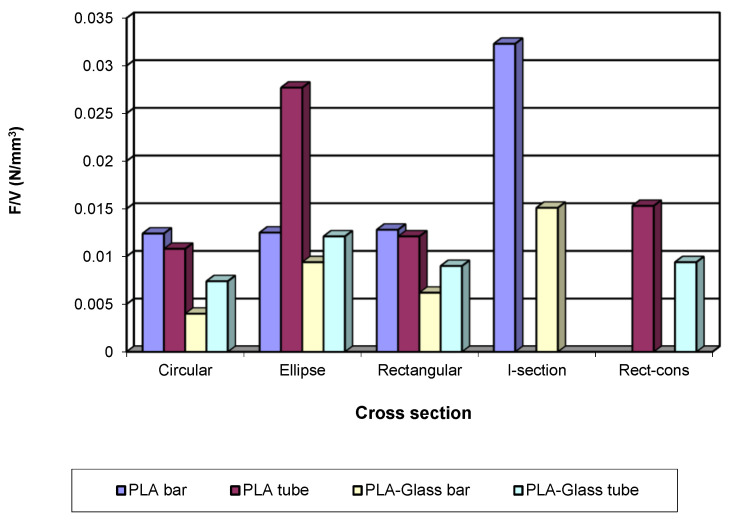
Efficiency of section geometry.

The difference between the test results and those of the simulation for bending strength was between—2.0% and 7.50% for the bar-type specimens and between—1.80% and 8.80% for the tube-type specimens. For the deformations, the differences were between −32.80% and 16.30% for the bar-type specimens and between −4.50% and 51.50% for the tube-type specimens. In the deformation, the value of the errors was higher; this was firstly due to the approximations of the deformed average fibre and, secondly, to the higher elasticity of the 3D printed structures of the tube-type. Furthermore, some deformation values were very small (2.60 mm), which may have led too error. For an easier and correct understanding of the geometrical efficiency, the results from Table 3 (last column) are presented in graphical form (see Figure 9).

## 4. Conclusions

Three-dimensional printing is a relatively new manufacturing process compared to the processes used by humans since antiquity, or even earlier. The novelty of the process did not prevent it from being implemented in many fields of goods production. The optimization of structures processed by different methods has become a necessity as the engineering sciences have developed, and computational technology has increased its computing capacity and speed. Through optimization, the material consumption decreases, while the geometry of the processed structures receives a shape that makes it possible to increase their loading capacity.

The presented study demonstrates that by applying the simulation process, it is possible to optimize the geometry of the section for 3D printed structures. To check whether the optimization by simulation of the geometry can be applied or not, several geometries of the section, several types of structures (bar, tube) and two materials were chosen. In this study, it was demonstrated that the use of simulation in the optimization process leads to obtaining results in line with those determined by tests.

This study demonstrates that the simulation process provides results close to those of tests and in line with the results presented in previously published. In some cases, the values of the deformations evolved inappropriately evolution, but upon analysis, it was possible to identify the causes. Consequently, by improving the modeling process, the number of errors can be diminished. For the bar-type specimens, from the bending resistance point of view, the most advantageous section was profile I and, after this, the elliptical section. For the tube-type specimens, the most advantageous section was the elliptical, followed by the rectangular section with consolidation. As mentioned, the results of the simulation process demonstrate similar results for the bending strength to those of the tests; however, in the area of force application, there was an increase in deviations compared to the values obtained through the tests. A possible solution is the restoration of the simulations but using a digital replica the elements that interact during the tests that is as accurate as possible. More specifically, this step means moving from a schematic to a more complex representation of the elements involved in the bending test (support and loading nose/anvil).

When the mechanical properties of 3D printed structures are known, the simulation process can be applied with good results in order to optimize the geometry of those structures. Depending on the progress that is registered in the field of simulation programs, as well as in the theory of the materials’ strength, the differences highlighted in this paper will possibly be reduced. Furthermore, other studies may yield new information on mechanical and technological properties, depending on the printing parameters, which will help to improve the simulation process; more precisely the differences between the simulation results and those of the tests will be reduced.

## Figures and Tables

**Table 1:** Specimen characteristics and codification (coding).

Filament Type	Cross Section	Dimensions (mm)	Specimen Type	Specimen Code
PLA	Circular	12	Bar	P_12
PLA-Glass	Circular	12	Bar	G_12
PLA	Circular	12×10	Tube	P_12_T
PLA-Glass	Circular	12×10	Tube	G_12_T
PLA	Ellipse	18×8	Bar	P_E18
PLA-Glass	Ellipse	18×8	Bar	G_E18
PLA	Ellipse	18×16	Tube	P_E18_T
PLA-Glass	Ellipse	18×16	Tube	G_E18_T
PLA	Rectangular	12.6×9	Bar	P_R12
PLA-Glass	Rectangular	12.6×9	Bar	G_R12
PLA	Rectangular	12.6×10.8	Tube	P_R12_T
PLA-Glass	Rectangular	12.6×10.8	Tube	G_R12_T
PLA	I-section	11.2×28	Bar	P_IS
PLA-Glass	I-section	11.2×28	Bar	G_IS
PLA	Rect-cons	12.6×10.8	Tube	P_RC_T
PLA-Glass	Rect-cons	12.6×10.8	Tube	G_RC_T

**Table 2:** Comparison between results of the tests and simulation, for 3D printed specimens, bending-stressed.

Specimen Cod	Test Results		Simulation Results	
	Strength (MPa)	Deformation (mm)	Strength (MPa)	Deformation (mm)
P_12	81.70	15.20	81.80	14.40
G_12	26.50	6.20	27.60	4.10
P_12_T	40.10	7.50	39.40	5.90
G_12_T	28.90	7.80	30.40	3.80
P_E18	54.90	5.80	55.30	6.10
G_E18	41.40	4.10	42.50	4.50
P_E18_T	82.30	6.40	83.10	8.20
G_E18_T	36.10	3.40	36.50	3.20
P_R12	62.70	9.10	61.50	9.70
G_R12	29.10	6.00	29.30	4.80
P_R12_T	35.50	4.60	38.60	5.20
G_R12_T	26.60	6.50	28.90	4.80
P_IS	45.70	4.20	47.80	4.90
G_IS	20.80	2.60	22.30	2.90
P_RC_T	32.10	5.70	33.80	6.80
G_RC_T	19.70	2.60	20.10	3.70

**Table 3:** Efficiency of the specimen cross-section.

Specimen Code	Cross Section	Surface (mm^2^)	Volume (mm^3^)	W_z_ (mm^3^)	W_z_/S (mm)	F/V (N/mm^3^)
P_12	Circular	113	24,900	170	1.50	0.012
G_12	Circular	113	24,900	170	1.50	0.004
P_E18	Ellipse	113	24,900	254	2.20	0.013
G_E18	Ellipse	113	24,900	254	2.20	0.009
P_R12	Rectangular	113	24,900	238	2.10	0.013
G_R12	Rectangular	113	24,900	238	2.10	0.006
P_12_T	Circular	34.50	7600	88	2.50	0.010
G_12_T	Circular	34.50	7600	88	2.50	0.007
P_E18_T	Ellipse	34.50	7600	115	3.30	0.028
G_E18_T	Ellipse	34.50	7600	115	3.30	0.012
P_R12_T	Rectangular	34.50	7600	116	3.40	0.012
G_R12_T	Rectangular	34.50	7600	116	3.40	0.009
P_IS	I-section	125	27,600	902	7.20	0.032
G_IS	I-section	125	27,600	902	7.20	0.015
P_RC_T	Rect-cons	71.70	8100	174	2.40	0.015
G_RC_T	Rect-cons	71.70	8100	174	2.40	0.009
